# Synergistic electrodeposition of bilayer films and analysis by Raman spectroscopy

**DOI:** 10.3762/bjoc.14.191

**Published:** 2018-08-21

**Authors:** Saadeldin E T Elmasly, Luca Guerrini, Joseph Cameron, Alexander L Kanibolotsky, Neil J Findlay, Karen Faulds, Peter J Skabara

**Affiliations:** 1 WestCHEM, Department of Pure and Applied Chemistry, University of Strathclyde, 295 Cathedral Street, Glasgow, G1 1XL, UK; 2 Current address: Chemistry Department, Faculty of Arts and Science (Tobruk), Omar Al-Mukhtar University, 919 El-Beida, Libya; 3 Current address: Department of Physical and Inorganic Chemistry and EMaS, Universitat Rovira i Virgili, Carrer de Marcel·lí Domingo s/n, 43007 Tarragona, Spain; 4 WestCHEM, School of Chemistry, Joseph Black Building, University of Glasgow, University Place, Glasgow, G12 8QQ, UK; 5 Institute of Physical-Organic Chemistry and Coal Chemistry, 02160 Kyiv, Ukraine

**Keywords:** bilayer, electropolymerisation, PEDOT, PEDTT, Raman

## Abstract

A novel methodology towards fabrication of multilayer organic devices, employing electrochemical polymer growth to form PEDOT and PEDTT layers, is successfully demonstrated. Moreover, careful control of the electrochemical conditions allows the degree of doping to be effectively altered for one of the polymer layers. Raman spectroscopy confirmed the formation and doped states of the PEDOT/PEDTT bilayer. The electrochemical deposition of a bilayer containing a de-doped PEDTT layer on top of doped PEDOT is analogous to a solution-processed organic semiconductor layer deposited on top of a PEDOT:PSS layer without the acidic PSS polymer. However, the poor solubility of electrochemically deposited PEDTT (or other electropolymerised potential candidates) raises the possibility of depositing a subsequent layer via solution-processing.

## Findings

Fabrication of multilayer organic electronic devices has been extensively researched in the past 20 years, resulting in numerous processes and techniques [1]. Recent advances include ink-jet printing [2] and direct stamping of the active layer to the substrate [3]. However, such processes involve the use of solvents, which can lead to blending of layers through dissolution of the initial layer [4]. While the use of water or fluorinated solvents can avoid these issues [5–6], materials suitable for use in such solvents are specifically designed, meaning such processes are less suitable for general use [4]. In this work, we present an alternative process for the fabrication of multilayer organic electronic devices. By electrochemically polymerising two different monomers in a step-wise fashion, a PEDOT/PEDTT bilayer was fabricated. Crucially, this approach provides an insoluble, conductive PEDOT layer, allowing the second PEDTT layer to be deposited on top (and subsequently de-doped), without compromising the initial deposition. Alemán and co-workers demonstrated this technique through the manufacture of multilayer films [7–8], alternating PEDOT and poly(*N*-methylpyrrole) to develop symmetric supercapacitors [9]. However, in their work there was no attempt to de-dope the second layer, which is necessary for the electrochemical preparation of a hole injection-semiconductor bilayer for solution processing of subsequent layers.

**Figure 1:**
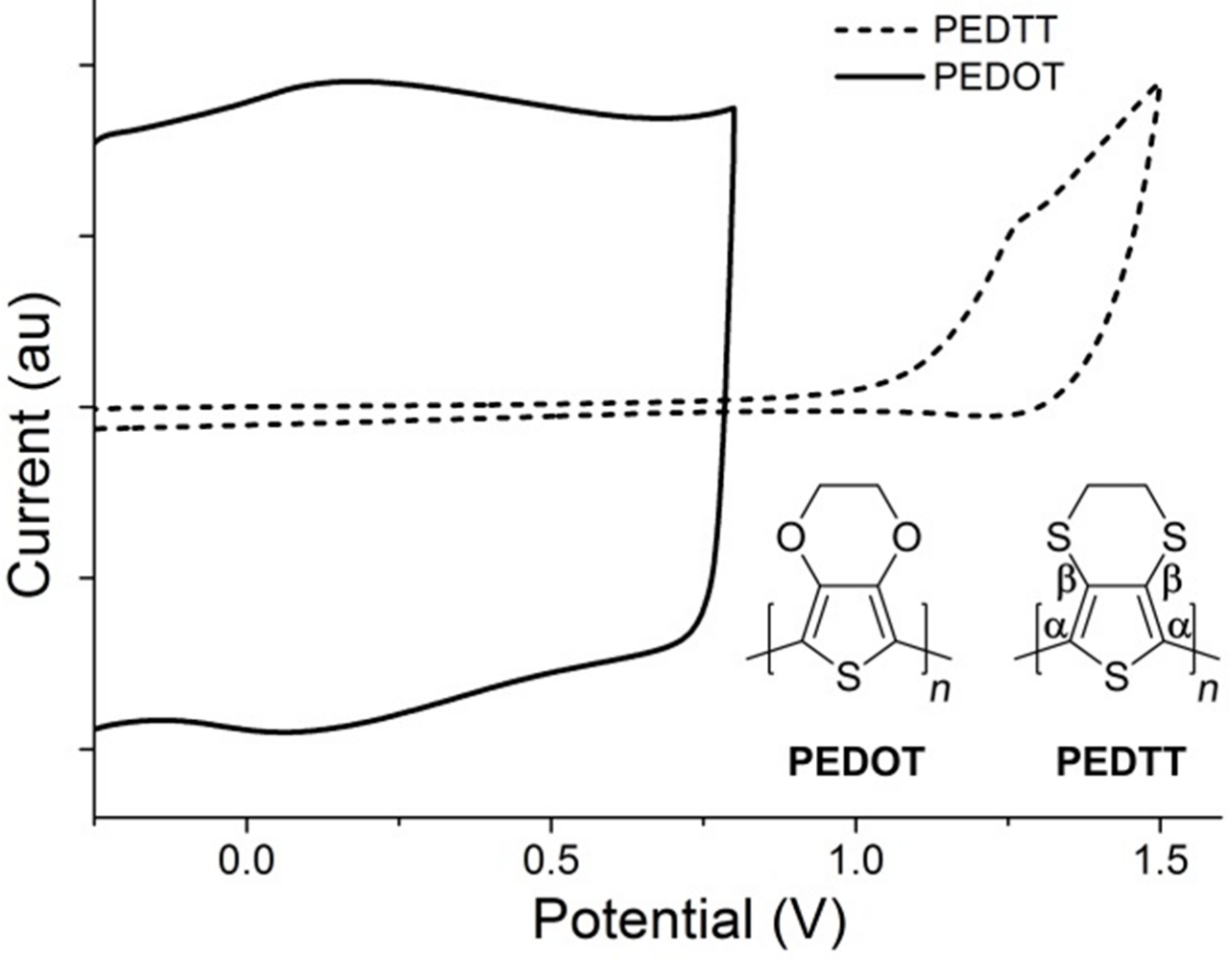
Oxidative wave for PEDOT (black line) and PEDTT (dashed line), together with the corresponding structures.

The monomers used in this work, 3,4-ethylenedioxythiophene (EDOT) and 3,4-ethylenedithiothiophene (EDTT), were either purchased commercially or prepared according to literature procedures [10]. Both were chosen due to their ease of electropolymerisation, excellent film-forming properties and because of their likely compatibility with each other [11]. First, PEDOT was deposited on an ITO glass slide by electropolymerisation of EDOT, using a Pt gauze counter electrode and a Ag wire quasi-reference electrode, with cycling between 0 V and +1.4 V over 150 cycles sufficient to achieve good polymer growth. Under similar conditions, the PEDOT/PEDTT bilayer was achieved using EDTT and cycling over the range +0.3 V to +1.78 V over 150 cycles (see Figure S1 in Supporting Information File 1 for the electropolymerisation of the PEDOT and PEDTT layers on a glassy carbon electrode). UV–vis absorption studies for the p-doped bilayer, carried out using ITO glass as the substrate, indicated that the main π–π* peak in the region of 450 nm was diminished, as expected for doped PEDOT and PEDTT (Figure S4a, Supporting Information File 1) [11]. The newly formed bilayer was de-doped by cycling between −0.5 V and −0.3 V over 300 segments in CH_3_CN (see Figures S2 and S3 in Supporting Information File 1 for the oxidation and reduction waves after dedoping). The UV–vis absorption spectrum of the de-doped bilayer clearly shows the signature of oxidised PEDOT with a broad absorption band in the region of 650–1000 nm (Figure S4b, Supporting Information File 1), similar to that previously reported [11]. Additionally, the λ_max_ of the de-doped bilayer corresponds to that of de-doped PEDTT [11] whilst there is no obvious peak at 580 nm, which would be expected if there was a significant amount of de-doped PEDOT present [11]. The absorption profile of the bilayer therefore clearly shows the selective de-doping of PEDTT. It is evident from Figure 1 that the two polymers show electroactivity in distinctly different potential windows. This has two consequences: (i) during the polymerisation of PEDTT, PEDOT remains doped and therefore conductive, allowing the polymerisation of EDTT to proceed; (ii) PEDTT can be de-doped within the electroactive window of PEDOT, meaning that the all-sulfur polymer can be successfully de-doped whilst the PEDOT layer remains predominantly doped.

**Figure 2:**
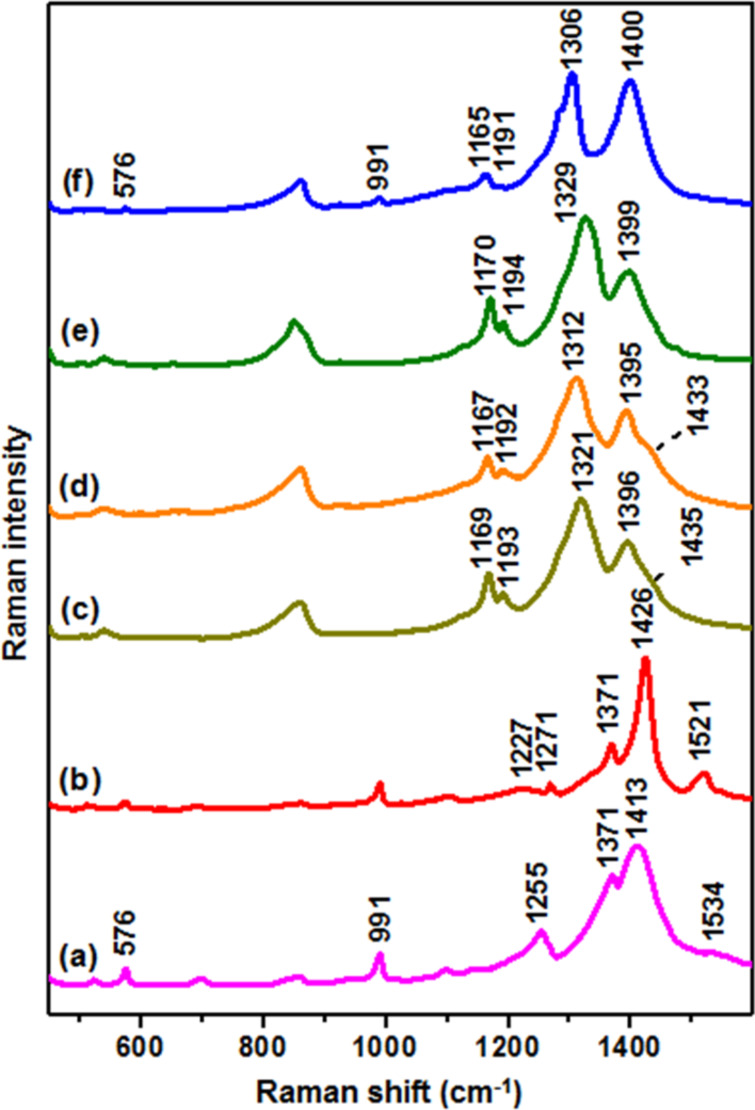
Normalised Raman spectra of (a) doped PEDOT monolayer; (b) de-doped PEDOT monolayer; (c) doped PEDTT monolayer; (d) de-doped PEDTT monolayer; (e) doped PEDOT/PEDTT bilayer; (f) de-doped PEDOT/PEDTT bilayer.

In order to support the formation of a PEDOT/PEDTT bilayer using this technique and to clarify the nature of doping in the two layers, freshly fabricated bilayers (using 10^−4^ M monomer solution) were grown on ITO and analysed by Raman spectroscopy, alongside doped and de-doped mono-layers of PEDOT and PEDTT for comparison. Figure 2 shows the Raman spectra of pure PEDOT and PEDTT polymers, both in the doped and neutral states (Figure 2a and b, Figure 2c and d, respectively). There are two main spectral regions of interest in the Raman spectra of these polymers. Below approximately 1150 cm^−1^, the Raman spectra are dominated by medium to weak bands which can be predominantly assigned to out-of-plane deformations [12], which are only weakly sensitive to changes in the electronic properties of the polymers [13]. In comparing PEDOT and PEDTT, contributions relating to the dioxyethylene ring of PEDOT, such as the bands at 576 cm^−1^ and 991 cm^−1^, assigned to dioxyethylene ring deformations, and at 1099 cm^−1^, assigned to C–O–C deformation, are absent in PEDTT (Figure 2c and d) [14].

On the contrary, the Raman features appearing above 1150 cm^−1^ are strongly dependent on the π-electron delocalisation within the polymer and, therefore, produce dramatic changes both in frequency and intensity due to the different electronic structure of each polymer. In particular, in the p-doped PEDOT spectrum (Figure 2a), Raman features corresponding to the thiophene ring at 1534 cm^−1^ (C=C asymmetric stretching vibration), 1413 cm^−1^ (symmetric C_α_=C_β_ stretching mode), 1371 cm^−1^ (C_β_=C_β_ stretching vibration), and 1255 cm^−1^ (inter-ring C_α_=C_α’_ stretching) are present [12]. When PEDOT is subjected to a de-doping process (Figure 2b) yielding the neutral polymer, the weak band at 1534 cm^−1^ shifts to 1521 cm^−1^ and increases in relative intensity, while the ν_sym_(C=C) band shifts from 1413 cm^−1^ to 1426 cm^−1^. Additionally, the broad band at 1255 cm^−1^ resolves into two distinct features (at 1227 and 1271 cm^−1^) [15] and a strong enhancement and sharpening of the Raman bands at 1371 and 1413 cm^−1^ is observed. Similar changes were ascribed by Garreau et al. [12] to the resonant effect of the Raman scattering. The marked intensity increase of the PEDOT Raman spectrum upon the de-doping process further supports this hypothesis (Figure S5, Supporting Information File 1).

By analogy with the band assignment for PEDOT, the Raman features in the PEDTT spectrum (Figure 2c) at 1396 cm^−1^ and 1321 cm^−1^, can be ascribed to ν_s_(C_α_=C_β_) and ν_s_(C_β_=C_β_) vibrations, respectively. However, the out-of-plane bands appearing in the region below 1150 cm^−1^ are less sensitive to the de-doping process, but the C=C bands show important changes, such as the marked downshift of the C_β_=C_β_ band from 1321 cm^−1^ to 1312 cm^−1^, and the relative intensity increase of the C_α_=C_β_ band at ≈1396 cm^−1^. In particular, Kocharova et al. [16] associated the intensity increase of the C_α_=C_β_ band in polythiophene structures to the higher localised charge at the C_α_=C_β_ linkage as a consequence of the positively charged doping of the material.

Figure 2e and 2f illustrate the Raman spectra of the polymer bilayer in the doped state and after de-doping of the PEDTT layer, respectively. The doped bilayer clearly shows the characteristic Raman profile of doped PEDTT, proving the effective and successful coating of the underlying PEDOT layer (Figure 2e). Once the bilayer is subjected to the de-doping process (Figure 2f), the Raman spectrum retains the spectral features of the PEDTT neutral polymer (Figure 2d). Very weak bands attributed to the PEDOT system, such as bands at 576 and 991 cm^−1^, can be recognised in the bilayer spectrum. The appearance of signals arising from the underlying PEDOT layer, although distant from the focal point of the laser, is likely the result of a partial de-doping of the underlying PEDOT layer. Thus, the much higher Raman scattering efficiency of PEDOT in the neutral state, as compared to PEDTT (Figure S5, Supporting Information File 1), enables the spectral emergence of these features [17]. Whilst it is not possible to identify the absorption of PEDTT in the broad absorption spectrum of the doped bilayer (Figure S4a, Supporting Information File 1), it has been shown that Raman spectroscopy is an effective technique to confirm the coating of PEDTT onto the doped PEDOT layer. Additionally, the presence of features that are characteristic of neutral PEDTT in the Raman spectrum of the de-doped bilayer confirms de-doping of PEDTT, complementing UV–vis absorption results (Figure S4b, Supporting Information File 1).

## Conclusion

In summary, we have shown a novel processing methodology for the fabrication of multilayer organic electronic devices that utilises electrochemical polymerisation to form the first two layers. Successful PEDOT/PEDTT bilayer formation has been confirmed by Raman spectroscopy. Electrodeposition of the bilayer has advantages over traditional processing methods including avoiding the acidity of PSS which is detrimental to the lifetime of devices containing PEDOT:PSS [18] and the ability to use polymers without insulating alkyl chains. It is important to note that the PEDTT layer can be substituted by any electropolymerised material that has a complementary electroactive potential window. Moreover, the insolubility of neutral PEDTT (or any other suitable polymer chosen) allows the bilayer to be subjected to solution-processing. It is therefore possible to deposit any layer onto the neutral insoluble polymer (e.g., an acceptor material for organic photovoltaics or an electron transport material for OLEDs) illustrating the potential for electrochemically deposited bilayers to be used for the fabrication of complex, multilayer organic electronic devices.

## Supporting Information

File 1 General experimental and additional spectra.
