# Supplementary material for: Synergistic electrodeposition of bilayer films and analysis by Raman spectroscopy
Source: Beilstein J Org Chem. 2018 Aug 21;14:2186–9. doi: 10.3762/bjoc.14.191 (PMC6122304; doi:10.3762/bjoc.14.191)
Supplement: File 1 — General experimental and additional spectra. [file Beilstein_J_Org_Chem-14-2186-s001.pdf]

Supporting Information

for

Synergistic electrodeposition of bilayer films and analysis by Raman spectroscopy

Saadeldin E. T. Elmasly[1,2], Luca Guerrini[1,3], Joseph Cameron[4], Alexander L. Kanibolotsky[4,5], Neil J. Findlay[4], Karen Faulds[1] and Peter J. Skabara[4]*

Address: [1]WestCHEM, Department of Pure and Applied Chemistry, University of Strathclyde, 295 Cathedral Street, Glasgow, G1 1XL, UK, [2]Current address: Chemistry Department, Faculty of Arts and Science (Tobruk), Omar Al-Mukhtar University, 919 El-Beida, Libya, [3]Current address: Department of Physical and Inorganic Chemistry and EMaS, Universitat Rovira i Virgili, Carrer de Marcel lí Domingo s/n, 43007 Tarragona, Spain, [4]WestCHEM, School of Chemistry, Joseph Black Building, University of Glasgow, University Place, Glasgow, G12 8QQ, UK and [5]Institute of Physical-Organic Chemistry and Coal Chemistry, 02160 Kyiv, Ukraine

Email: Peter Skabara - peter.skabara@glasgow.ac.uk

*Corresponding author

General experimental and additional spectra

Contents

- General experimental
- Figure S1 – Electropolymerisation of PEDOT and PEDTT layers
- Figure S2 – Oxidation profile of PEDOT/PEDTT bilayer
- Figure S3 – Reduction profile of PEDOT/PEDTT bilayer
- Figure S4 – UV–vis absorption profile of PEDOT/PEDTT bilayer
- Figure S5 – Raman spectra

General experimental

Cyclic voltammetry (CV) measurements and electropolymerisations were performed on a CH Instruments 660A electrochemical workstation with *iR* compensation using anhydrous dichloromethane or acetonitrile as the solvent. The electrodes were glassy carbon (or ITO glass), platinum wire and silver wire as the working, counter and quasi-reference electrodes, respectively. All solutions were degassed (Ar) and contained monomer substrates in concentrations of ca. 10^{-4} M, together with n-Bu_4NPF_6 (0.1 M) as the supporting electrolyte. All measurements are referenced against the $E_{1/2}$ of the Fc/Fc^+ redox couple. Absorption spectra were recorded on a Unicam UV 300 instrument.

Raman spectra were acquired using a Renishaw *System 2000* Raman spectrometer equipped with a 785 nm excitation line and ×50/0.75 Leica objective. A ≈8 mm × 20 mm polymer layer deposited over an ITO slide has been investigated by Raman spectroscopy, collecting individual spectra in 15 randomly located points over the slide. The Raman signal appeared substantially unaltered over all investigated areas for all polymers, indicating the high homogeneity of materials (spectra not shown). The Raman spectra presented in this paper are the average signal obtained from these 15 different spots.

a)

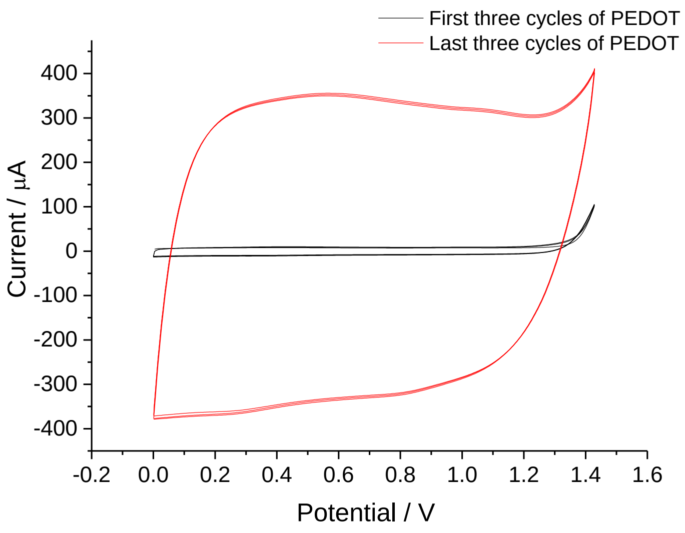

b)

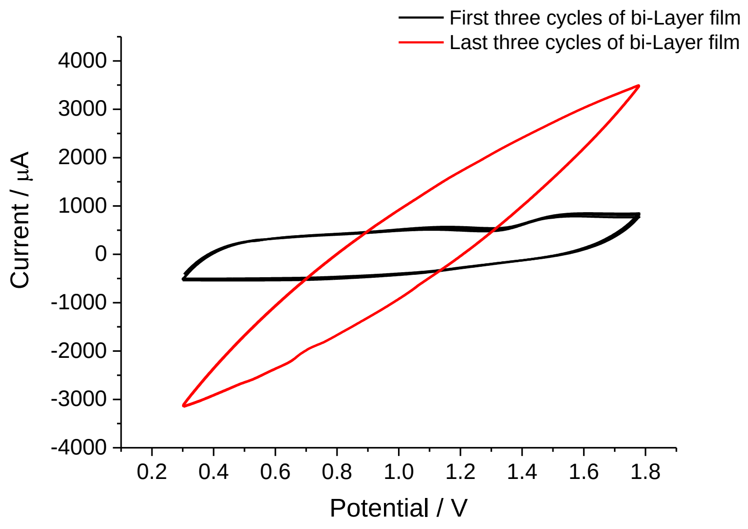

Figure S1: Compilation graph showing the cyclic voltammograms for the electropolymerisation of a) PEDOT and b) PEDTT to form bilayer onto a glassy-carbon working electrode. For clarity, the first three and the last three scans are shown only.

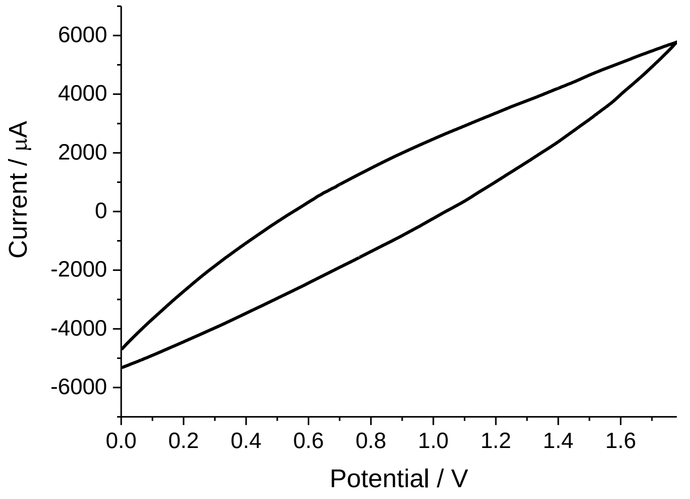

Figure S2: Oxidation profile of the PEDOT/PEDTT bilayer. Oxidation profile of PEDOT/PEDTT bilayer using glassy carbon working electrode, Ag wire quasi-reference electrode and Pt counter electrode in acetonitrile (0.1 M $TBAPF_6$ as supporting electrolyte) with a scan rate of 100 mV s^{-1}.

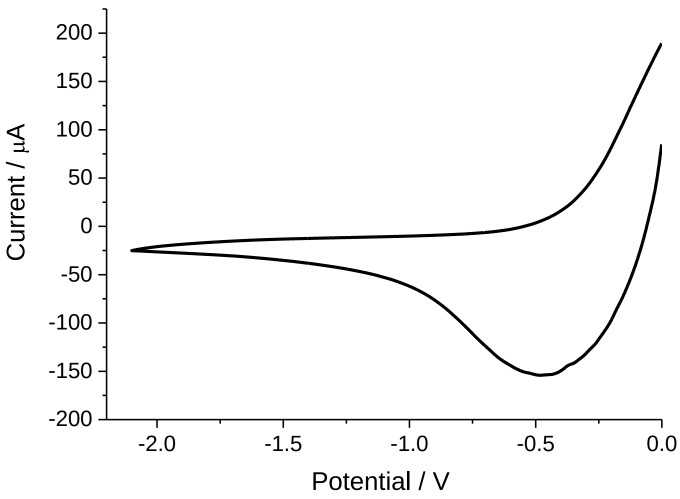

Figure S3: Reduction profile of the PEDOT/PEDTT bilayer. Reduction profile of PEDOT/PEDTT bilayer using glassy carbon working electrode, Ag wire quasi-reference electrode and Pt counter electrode in acetonitrile (0.1 M $TBAPF_6$ as supporting electrolyte) with a scan rate of 100 mV s^{-1}.

a)

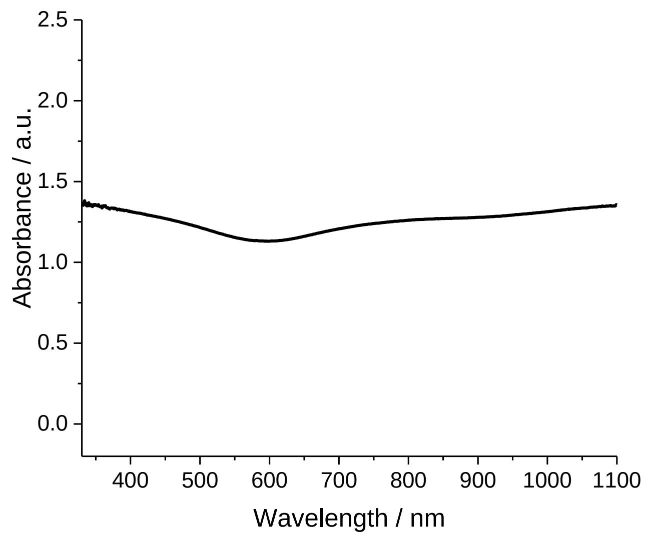

b)

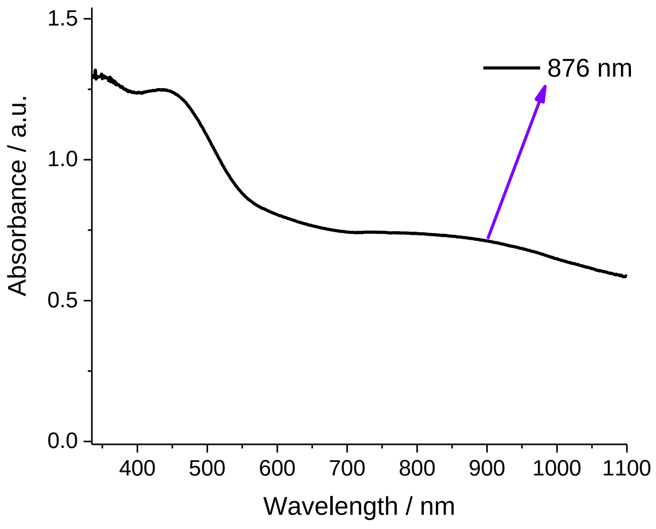

Figure S4: UV–vis absorption profile of PEDOT/PEDTT bilayer with PEDTT a) doped and b) de-doped. Absorption spectrum of a poly(PEDOT/PEDTT) bilayer as a thin film on ITO-coated glass prepared in monomer solution with acetonitrile, de-doped between −0.5 to −0.3 V over 300 segments.

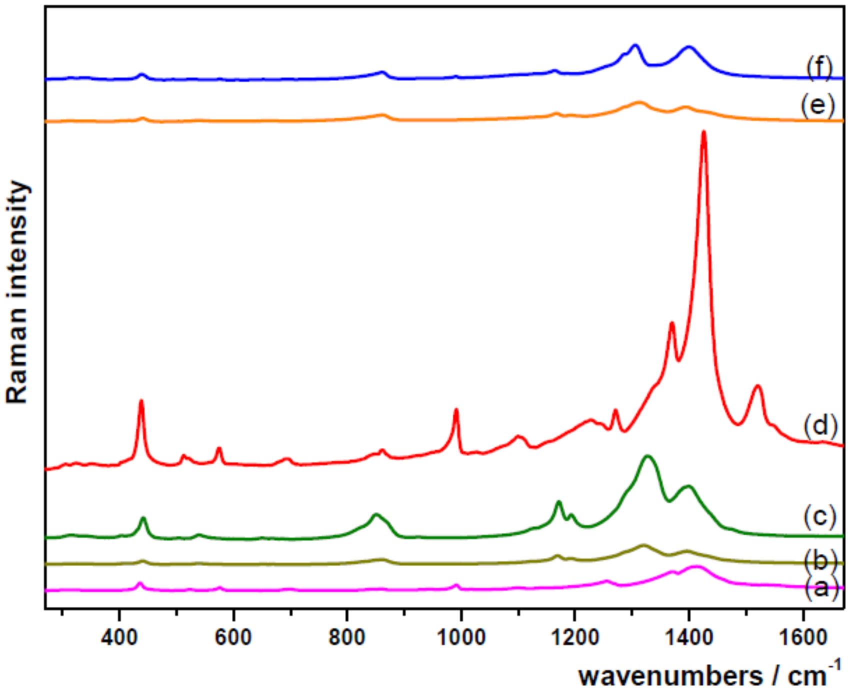

Figure S5: Raman spectra of (a) PEDOT doped, (b) PEDTT doped, (c) bilayer doped, (d) PEDOT de-doped, (e) PEDTT de-doped and (f) bilayer de-doped.
